# Brace yourselves, vaccine-preventable diseases are coming! The impact of war on polio and other vaccine-preventable diseases

**DOI:** 10.17179/excli2024-8022

**Published:** 2025-01-03

**Authors:** Thalles Guilarducci Costa, Rizia Rocha-Silva, João Victor Rosa de Freitas, Bráulio Evangelista de Lima, Rodrigo Luiz Vancini, Marília Santos Andrade, Claudio Andre Barbosa de Lira

**Affiliations:** 1 State University of Goiás, University Unit of Itumbiara; 2 Faculty of Physical Education and Dance, Federal University of Goiás, Goiânia, Brazil; 3 Center for Physical Education and Sports, Federal University of Espírito Santo, Vitória, Brazil; 4 Department of Physiology, Federal University of São Paulo, São Paulo, Brazil

## ⁯⁯⁯⁯

Recently, Israeli Prime Minister Benjamin Netanyahu agreed to a humanitarian pause in the Gaza war to allow children to be vaccinated against polio (Devi, 2024[7]). The aim was to vaccinate around 640,000 children throughout the Gaza Strip. This action comes after the detection of highly infectious wild poliovirus in sewage samples in June and after a 10-month-old baby was partially paralyzed due to the polio diagnosis. This case is the first after 25 years without any registered polio cases in Gaza territory (Devi, 2024[7]).

Previous manuscripts warned about the risk of polio and other disease resurgences, driven by vaccine hesitancy and unequal immunization coverage (Dubé et al., 2013[9]; Costa et al., 2023[6]). Nowadays, this risk is even higher; but the main cause is different: war. War is one of the most challenging emergencies, marked by the disruption of essential social functions, including access to healthcare. In these contexts, the healthcare system is forced to prioritize the immediate treatment of the wounded and sick, and the goal of disease prevention becomes secondary, further increasing the vulnerability to outbreaks of preventable diseases such as polio (Obradovic et al., 2014[14]; Armitage, 2022[2]).

Furthermore, basic immunizations are often missed in conflict-affected areas due to the collapse, loss of healthcare workers, and the deliberate destruction of vital health facilities (Grundy and Biggs, 2018[10]). Tertiary healthcare tends to be prioritized over primary healthcare. Among the various challenges to providing primary healthcare to the population, the storage of vaccines and other temperature-sensitive medicines becomes especially challenging during war, as these items must be kept cold to remain effective. 

Moreover, displaced populations, including refugees and internally displaced persons, often live in overcrowded conditions in informal urban settlements, reception centers, camps or immigration detention centers, where infectious diseases can easily spread. It is important to note that these displaced populations often face barriers in accessing routine healthcare services, including vaccines, due to a lack of civil documentation and registration.

Unfortunately, the situation faced in Gaza is not an isolated incident. Similar disruptions to public health efforts can be seen in other conflict zones worldwide. Other wars have caused the collapse of health systems and immunization coverage. For instance, in Yemen, a country embroiled in civil war since 2015, a diphtheria outbreak occurred between 2017 and 2018 with 2,203 reported cases and 116 deaths (Al-Dar et al., 2022[1]). This outbreak reflects the gap in vaccine coverage over the previous three years, due to the collapse of the health system amid the war. Diphtheria is a life-threatening disease that can lead to upper airway obstruction, among other complications (Blumberg et al., 2018[5]). However, it is preventable by a pentavalent vaccine, which also covers four other diseases, tetanus, pertussis, hepatitis B, and *Haemophilus* *influenzae* type B, and is administered in three doses, at 6, 10, and 14 weeks of age (WHO, 2012[23]). A descriptive study in Yemen showed, in addition to diphtheria, a drop in vaccine coverage for measles and Bacillus Calmette-Guérin (BCG) after two years of war, indicating that the country was facing increasing risk of vaccine-preventable disease outbreaks (Torbosh et al., 2019[20]).

In Ukraine, 6,739,400 of its citizens have already become refugees worldwide since the Russian invasion (Operational Data Portal, 2024[17]). The country is currently facing low vaccination rates, in addition to the imminent collapse of its healthcare systems and limited access to humanitarian assistance due to widespread insecurity (Holt, 2024[11]). In a similar context, COVID-19 vaccination rates were already suboptimal before the war and have deteriorated further with the Russian invasion (Awuah et al., 2022[4]).

Although the World Health Organization declared over the vaccine-derived type 2 poliovirus outbreak that began in October 2021, in September 2023, experts continue to warn of ongoing challenges to immunization efforts. Type 2 poliovirus is closely linked to the incomplete administration of the immunization regimen, which requires multiple doses to be effective. When the immunization regimen is not completed, the attenuated poliovirus (present in the vaccine) can evolve during replication, regaining its wild-type characteristics i.e., its virulence and transmissibility potentially leading to cases of vaccine-associated paralytic poliomyelitis (Dowdle et al., 2003[8]). In this context, two circulating vaccine-derived poliovirus type 2 cases were reported in Ukraine in 2022 (WHO, 2022[22]). 

A relevant consequence of any war is the intense migratory flow generated. European countries that receive these refugees are facing public health challenges due to the large influx of people fleeing war (Lewtak et al., 2022)[12]. Furthermore, there are over 5 million refugees or asylum-seekers from the Afghanistan war, living in Iran, Tajikistan, Uzbekistan, Turkmenistan and Pakistan. Despite the Afghanistan war ending in 2021, with the withdrawn of United States troops, the country continues to suffer with Taliban occupation (Operational Data Portal, 2024[15]). Other cases like Somalia's civil war, led to 760,000 refugees in Africa's Horn (Operational Data Portal, 2024[16]). A total of 1.5 million displaced Yemenis from war are settled in camps with low infrastructure, without access to the minimum level of basic services (Operational Data Portal, 2024[18]). Therefore, this is not just a localized issue; it poses a global threat because refugees fleeing violence can carry pathogens across borders, triggering new outbreaks far from the original conflict zones. Taken together, this data is deeply concerning and underscores the urgent need for coordinated efforts by authorities to prevent the resurgence of vaccine-preventable diseases.

Individuals of lower socioeconomic status are particularly vulnerable in conflict zones, as they often lack the means to migrate to safer areas like their wealthier counterparts (Armitage, 2022[3]). According to the United Nations Children's Fund (UNICEF), approximately 40 % of unvaccinated or under-vaccinated children live in countries partially or entirely affected by conflict. These children are often the most susceptible to outbreaks of diseases such as measles and poliomyelitis, which can result in death or severe disability (UNICEF, 2024[21]).

Thus, from our perspective ensuring access to life-saving vaccines is not only a humanitarian imperative but also a crucial strategy to protect global public health. As conflicts continue to destabilize regions and displace millions of people, the collapse of health systems and the subsequent decline in vaccination coverage provide fertile ground for the resurgence of vaccine-preventable diseases. Therefore, strategies such as conducting security assessments, negotiating secure physical access, engaging local communities, coordinating humanitarian aid deliveries, implementing transit or cross-border vaccination strategies, and collaborating with the military or other security forces could reduce the morbidity and mortality of vaccine-preventable diseases in conflict settings (Nnadi et al., 2017[13]; Grundy and Biggs, 2018[10]).

The successful polio vaccination campaign in Gaza, supported by the United Nations amidst a fragile ceasefire (Reuters, 2024[19]), highlights critical approaches to overcoming immunization challenges in conflict zones. 

Therefore, to enhance vaccination efforts in conflict-affected regions, several strategies can be implemented:

- Establishing temporary ceasefires: Negotiating and securing humanitarian ceasefires with conflicting parties can create safe periods for vaccine delivery and administration.
- Leveraging local networks: Engaging trusted local healthcare workers and community leaders can improve the acceptance and reach of immunization campaigns. These actors bring valuable cultural and logistical insights, enhancing outreach and communication.
- Cross-sector coordination: Strengthening collaborations between international organizations, local governments, non-governmental organizations (NGOs), and humanitarian agencies ensures efficient resource utilization and minimizes operational redundancies.
- Innovative vaccine delivery methods: Utilizing strategies such as mobile vaccination units, transit vaccination points, and cross-border immunization corridors can effectively reach displaced populations and those in remote or high-risk areas.
- Community engagement and advocacy: Addressing vaccine hesitancy and misinformation through targeted educational campaigns can foster trust and improve public cooperation in affected communities.
- Strengthening health infrastructure: Partnering with international donors to rebuild or temporarily reinforce healthcare systems in conflict zones supports the sustainable delivery of immunization programs.
- Security collaborations: Coordinating with neutral security forces or peacekeeping missions can ensure the safety of healthcare workers during vaccine distribution efforts.
- By adopting these multifaceted approaches, the global community can work collectively to mitigate the devastating health impacts of armed conflicts and promote equitable access to life-saving vaccines.

## Funding

This research was financed in part by the Coordenação de Aperfeiçoamento de Pessoal de Nível Superior - Brasil (CAPES) - Finance Code 001. TGC, RRS, JVRF, and BEL are fellowships at CAPES. CABL and MSA are productivity fellowships at the Conselho Nacional de Desenvolvimento Científico e Tecnológico - Brasil (CNPq). RLV is a productivity fellowship at the Fundação de Amparo à Pesquisa do Estado do Espírito Santo - Brasil (FAPES).
